# The relative contribution of climate variability and vector control coverage to changes in malaria parasite prevalence in Zambia 2006–2012

**DOI:** 10.1186/s13071-016-1693-0

**Published:** 2016-08-05

**Authors:** Adam Bennett, Josh Yukich, John M. Miller, Joseph Keating, Hawela Moonga, Busiku Hamainza, Mulakwa Kamuliwo, Ricardo Andrade-Pacheco, Penelope Vounatsou, Richard W. Steketee, Thomas P. Eisele

**Affiliations:** 1 Malaria Elimination Initiative, Global Health Group, University of California, 500 16th St, San Francisco, CA 94158 USA; 2 Center for Applied Malaria Research and Evaluation, Tulane University School of Public Health and Tropical Medicine, New Orleans, LA USA; 3 PATH Malaria Control and Evaluation Partnership in Africa (MACEPA), Lusaka, Zambia; 4 National Malaria Control Centre, Ministry of Health, Lusaka, Zambia; 5 Swiss Tropical and Public Health Institute, Basel, Switzerland; 6 University of Basel, Basel, Switzerland

**Keywords:** Malaria, Climate, Vector control, Geostatistics

## Abstract

**Background:**

Four malaria indicator surveys (MIS) were conducted in Zambia between 2006 and 2012 to evaluate malaria control scale-up. Nationally, coverage of insecticide-treated nets (ITNs) and indoor residual spraying (IRS) increased over this period, while parasite prevalence in children 1–59 months decreased dramatically between 2006 and 2008, but then increased from 2008 to 2010. We assessed the relative effects of vector control coverage and climate variability on malaria parasite prevalence over this period.

**Methods:**

Nationally-representative MISs were conducted in April-June of 2006, 2008, 2010 and 2012 to collect household-level information on malaria control interventions such as IRS, ITN ownership and use, and child parasite prevalence by microscopic examination of blood smears. We fitted Bayesian geostatistical models to assess the association between IRS and ITN coverage and climate variability and malaria parasite prevalence. We created predictions of the spatial distribution of malaria prevalence at each time point and compared results of varying IRS, ITN, and climate inputs to assess their relative contributions to changes in prevalence.

**Results:**

Nationally, the proportion of households owning an ITN increased from 37.8 % in 2006 to 64.3 % in 2010 and 68.1 % in 2012, with substantial heterogeneity sub-nationally. The population-adjusted predicted child malaria parasite prevalence decreased from 19.6 % in 2006 to 10.4 % in 2008, but rose to 15.3 % in 2010 and 13.5 % in 2012. We estimated that the majority of this prevalence increase at the national level between 2008 and 2010 was due to climate effects on transmission, although there was substantial heterogeneity at the provincial level in the relative contribution of changing climate and ITN availability. We predict that if climate factors preceding the 2010 survey were the same as in 2008, the population-adjusted prevalence would have fallen to 9.9 % nationally.

**Conclusions:**

These results suggest that a combination of climate factors and reduced intervention coverage in parts of the country contributed to both the reduction and rebound in malaria parasite prevalence. Unusual rainfall patterns, perhaps related to moderate *El Niño* conditions, may have contributed to this variation. Zambia has demonstrated considerable success in scaling up vector control. This analysis highlights the importance of accounting for climate variability when using cross-sectional data for evaluation of malaria control efforts.

**Electronic supplementary material:**

The online version of this article (doi:10.1186/s13071-016-1693-0) contains supplementary material, which is available to authorized users.

## Background

Scale-up of vector control interventions, specifically ownership and use of insecticide-treated nets (ITNs) and indoor residual spraying (IRS), has been shown in multiple settings to reduce malaria morbidity and mortality [1–5]. Zambia made significant progress in scaling up ITN and IRS coverage from 2005 through 2012, with progress documented through repeated nationally-representative household surveys [6, 7]; over 10 million ITNs were distributed between 2005 and 2012, and roughly 850,000 structures were sprayed annually during this period. Nationally-representative Malaria Indicator Surveys (MIS) were conducted in 2006, 2008, 2010 and 2012 to monitor the progress in household coverage of ITNs and IRS at the population level, as well as to provide estimates of malaria parasite prevalence in children 1–59 months at these time-points.

Although household ownership and use of ITNs and IRS coverage as measured by these surveys increased nationally from 2006 through 2012, the proportion of children < 5 years old with a malaria parasite infection declined from 22.4 % in 2006 to 9.3 % in 2008 [8], but increased somewhat to 15.9 % in 2010 and 14.6 % in 2012. The increase between 2008 and 2010 was variable across the country and greatest in rural areas, nearly doubling in some provinces; the proportion of children with severe anemia increased in a similar pattern with parasitemia between 2008 and 2010, from 4.3 to 9.2 %.

Identifying possible causes of this observed resurgence in infection prevalence between 2008 and 2010 has broad implications for malaria control programs across Africa. A historical analysis of global occurrences of malaria resurgence suggests that in most cases a flagging of malaria control effort led to an increase in incidence rates [9]. There is growing evidence of potential ITN effectiveness decay in sub-Saharan Africa (SSA) due to insecticide resistance and changes in vector biting behavior [10–12]. There is further speculation that anomalous weather patterns-perhaps related to a relatively strong *El Niño* episode in late 2009 and early 2010-may have influenced this resurgence in transmission, as observed elsewhere [13, 14].

Weather variability is a well-known driver of malaria transmission [15]. High-resolution climate and environmental data are being used with increasing sophistication in geostatistical modeling frameworks for malaria risk mapping purposes [16], where the objective is usually the production of mean endemicity surfaces [17], incidence prediction [18] or to examine changes in malaria parasite prevalence over time [19, 20]. However, until recently climate data have seldom been directly incorporated into evaluations of program impact [21–23]. Three recent examples where climate data were successfully incorporated include the evaluation of vector control scale-up and incidence data in Eritrea by Graves and colleagues [21], work by Giardina and colleagues comparing changing parasite prevalence and vector control coverage in five countries [24], and a continental-scale evaluation by Bhatt and colleagues [5]. In order to obtain unbiased estimates of the impact of malaria control in program evaluations and assess any potential change in their effectiveness over time, analyses must directly incorporate changes in climate that influence malaria transmission potential.

Zambia reported increases in malaria parasite prevalence and health system reported clinical incidence between 2008 and 2010 that continued in 2012, despite continued scale-up of malaria control interventions during this time. This paper assessed the association between inter-annual climatic and other environmental variables, IRS and ITN coverage, and changes in malaria parasite prevalence between 2006, 2008, 2010 and 2012 while accounting for confounding factors at the subnational level. We used geostatistical models to estimate the relative contribution of subnational changes in IRS and ITN coverage and climate to changes in malaria parasite prevalence over this period.

## Methods

### Malaria indicator surveys

We used data from three Malaria Indicator Surveys (MISs), each of which was conducted at the end of the high transmission season between April and June in 2006, 2008, 2010 and 2012. The sampling design and questionnaire for these surveys has been described elsewhere [25]. Briefly, the sample size and standard enumeration areas (SEAs) were selected to provide precise estimates of ITN coverage at the national, provincial, and urban/rural levels. At the time of the surveys, there were 9 provinces and 72 districts in Zambia. While a new province was demarcated in 2011, we maintained the original 9 provinces for consistency in this analysis. A two-stage sampling design was used, with the primary sampling units consisting of standard enumeration areas (SEAs) selected proportional to the estimated population size (PPS) of each within provincial and urban/rural strata. Within each selected SEA, field workers conducted a complete household enumeration using personalized-digital assistants (PDAs) equipped with GPS, and selected 25 households for questionnaire administration to the household head and caregivers of children under 5 years of age. Latitude and longitude were collected for each household. For SEA-level geographic information, we determined the centroid for each SEA by averaging household latitudes and longitudes.

### Primary outcome

Malaria parasite prevalence in children 1–59 months served as the primary outcome. Malaria parasite prevalence was ascertained from infection status by quality controlled slide microscopy for all children 1–59 months in selected households during each MIS; HRP2 rapid diagnostic tests (RDTs) (ICT Malaria Pf) were used in the field to provide point of care diagnosis and treatment for children testing positive.

### Measures of explanatory variables and potential confounding factors

Publicly-available remote-sensing climate data referenced to the centroid of each selected survey SEA were compiled from the following sources: the African Data Dissemination Service for 10-day rainfall estimates at 8 km resolution [26], the USGS Hydro 1 k dataset for elevation at 1 km resolution [27], the MODIS satellite data repository for 16-day enhanced vegetation index (EVI) at 1 km resolution [28], the monthly Temperature Suitability Index (TSI) from the Malaria Atlas Project (MAP) [29], and Worldpop for population estimates at 1 km resolution [30]. We also investigated program administrative shape-files (*.shp*) for road networks, health facilities, and water bodies. Time-variant climate and environmental data (rainfall, TSI, and EVI) were extracted for the four months preceding the start of each survey at 1 km resolution.

In each MIS, a net roster was used to count the number of mosquito nets present in each selected household, including when the net was procured and who in the household slept under the net the previous night. If and when IRS was conducted was ascertained based on the recall of the head of household during each MIS. We defined a mosquito net as an ITN if it was a long-lasting insecticide-treated net (LLIN), or any net that had been insecticide treated within the past 12 months [31]. Both household and community-level ITN ownership were considered: for community-level coverage, we calculated the ITN density (number of ITNs per household members) for each SEA. We defined IRS as the survey respondent reporting that household walls were sprayed within the 12 months preceding the survey date. Similarly, we calculated the proportion of households having received IRS per SEA as a measure of community-level coverage.

As a measure of household socioeconomic status, principle-components analysis (PCA) was used to create a wealth index from a list of household assets captured during each survey [32]; this asset list did not include any household vector control items. Population density was calculated from the Worldpop raster and used as a measure of urban-ness. We classified areas with < 1,000 population per km^2^ as rural areas and areas with > 1,000 population per km^2^ as urban/peri-urban areas. This has been shown to be a reliable cut-off for stratifying malaria risk [33]. We also calculated the Euclidean distance from each household to the nearest water body, including permanent lakes and major rivers.

### Variable selection and statistical analysis

Descriptive comparisons of household vector control and parasite prevalence were conducted across surveys both nationally and by province, using survey-weighted point estimates.

#### Climate variables

For modeling and predicting parasite prevalence using time-varying climatic data, we considered both biological plausibility and statistical fit to select climatic variables based on lag periods preceding household data collection. For EVI, lags up to the beginning of survey data collection were considered as this index represents the effect of cumulative rainfall and humidity [further details of the selection of climate variables are presented in Additional file 1]. The best-fitting lags within biologically plausible time frames included in final models were the 20-day period of rainfall ending 6 weeks prior to the start of data collection, and the 16-day EVI in the week preceding the start of survey data collection.

#### Model selection

Bivariate and multivariable associations between the outcome of parasite infection and explanatory variables were assessed using random effects logistic regression models for each survey round. Linear, categorical, and non-linear relationships between the set of explanatory variables and parasite prevalence were assessed in separate models for each survey; interaction terms between survey year and ITN coverage, climate, and other covariates were assessed in final pooled models. Final non-spatial model selection was based on the Akaike’s Information Criterion (AIC); final spatial model selection was based on the Deviance Information Criterion (DIC). Model fit assessments were conducted in Stata 13.1 [34] and R 3.3.0 [35].

To account for spatial autocorrelation and to produce continuous prediction surfaces for examining spatial patterns in ITN coverage and parasite prevalence, final models were run in a Bayesian geostatistical framework with spatially-correlated random effects (further details in Additional file 1). We compared results of several different geostatistical models including individual, household, and SEA-level parameters to confirm that effect estimates used for final prediction models were not modified by individual level parameters. Additionally, we compared results of geostatistical models with non-spatial random effects models. In a model (‘full model’) including individual, household, and cluster level parameters we included individual and household-level socio-demographic and vector control parameters (ITNs and IRS), and SEA-level measures of vector control (ITNs and IRS), geography (elevation and Euclidean distance to nearest water body), and climate (rainfall and EVI). In the spatial prediction model (‘prediction model’) we included only the SEA-level ITN, IRS, geographic, and climate parameters. Additionally, to compare results of varying ITN and IRS coverage and climate inputs on predictions, we created separate geostatistical models of ITN coverage, defined as the ratio of ITNs:HH members, and IRS coverage for inclusion in final prediction models. All model parameters were estimated using Integrated Nested Laplace Approximation (INLA) in R [36] (Details in Additional file 1).

#### Model validation and prediction

We conducted validation tests on the spatial prediction model and a comparative non-spatial model by calibrating each model on an 85 % training dataset for predicting to the remaining 15 % of locations. The training and prediction datasets were selected through stratified sampling to ensure equal allocation by survey year. We calculated the mean prediction error (MPE) as a measure of bias and the root mean square error (RMSE) as a measure of accuracy. For the final prediction model surfaces, we plotted the posterior mean prediction values for each 5 km pixel (roughly 40,000 pixels covering Zambia) using INLA in R [36] and ArcGIS [37]. We evaluated several different prediction scenarios by including different combinations of climate and ITN and IRS coverage surfaces for each year as prediction model inputs. For alternative prediction scenarios we focused primarily on changes between 2008 and 2010 as this period was characterized by anomalous climate conditions and the largest changes in measured prevalence. For each prediction scenario we calculated final population-averaged parasite prevalence values per province by multiplying the mean posterior prediction raster by the Worldpop population raster for children < 5 to obtain the estimated number of infected children < 5 by province, and then dividing by the total province < 5 population. To approximate uncertainty in province level estimates, we calculated the population-averaged parasite prevalence for the 2.5 and 97.5 % posterior quantiles.

## Results

**Fig. 1:**
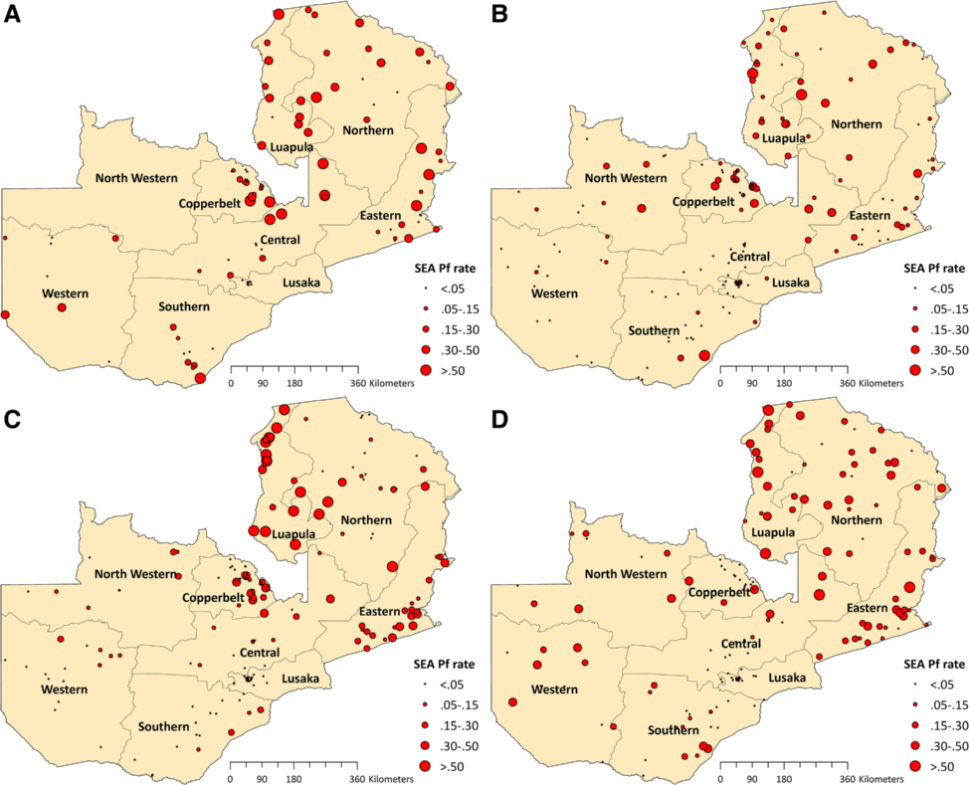
Sampled SEAs and SEA-level (cluster) *Plasmodium falciparum* parasite rate (*Pf*PR_1–59_) in **a** 2006, **b** 2008, **c** 2010, and **d** 2012

The 2006 MIS covered 2,889 households, with slide results available for 1,787 children 1–59 months of age in 109 SEAs (Fig. 1). The 2008 MIS covered 4,142 households, with slide results available for 3,010 children in 169 SEAs, while the 2010 MIS covered 4,361 households, with slide results available for 3,423 children in 178 SEAs. The 2012 MIS covered 3,800 households, with slide results available for 2,962 children in 158 SEAs.

**Fig. 2:**
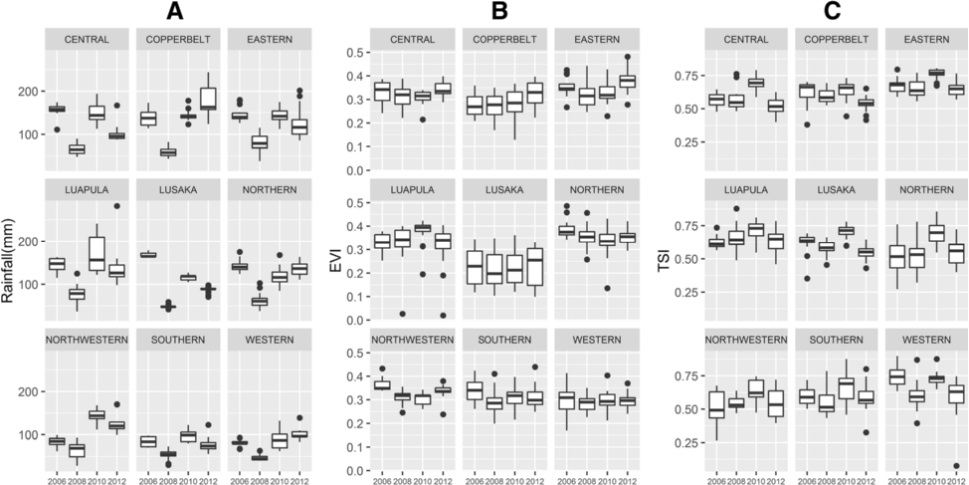
Box plots of SEA-level rainfall (**a**), enhanced vegetation index (**b**), and temperature suitability index (**c**) from best fitting periods preceding each survey, by province and year, Zambia 2006-2012

Rainfall at survey locations preceding each survey was lowest in 2008 and highest in 2010 across most provinces (Fig. 2); rainfall increased further in 2012 in Copperbelt and Western province. Similarly, temperature suitability preceding each survey was highest in 2010 in most provinces. EVI was highest in 2010 in Luapula, and slightly higher in 2010 than 2008 in Eastern province; EVI increased further in Eastern and Copperbelt provinces in 2012.

While national vector control coverage increased consistently from 2006 to 2008 across Zambia, changes in vector control coverage from 2008 to 2010 varied considerably by province, and differed for ITN and IRS coverage. Between 2008 and 2010, household ownership of ≥ 1 ITN fell significantly in Luapula (from 69.8 to 50.0 %, *P* < 0.05) and Northern provinces (from 90.9 to 61.2 %, *P* < 0.001), while IRS coverage increased significantly in both areas (Table 1); combined, the percent of households with any vector control (ITN and/or IRS) fell significantly in both provinces. In other provinces - Eastern, Copperbelt and Central, household ITN ownership increased slightly. Between 2010 and 2012, the percent of household with an ITN and/or IRS increased substantially in Luapula (from 60.9 to 91.4 %, *P* < 0.001), Eastern (from 77.8 to 90.4 %, *P* < 0.05), and Northern (from 66.1 to 78.6 %, *P* < 0.05), but decreased in Western (from 79.3 to 56.0 %, *P* < 0.05) and Central (from 77.7 to 65.2 %, *P* < 0.05).

**Table 1:** Survey measured percent of household owning ≥ 1 ITN and/or receiving IRS by year and province, Zambia 2006–2010

	ITN ownership (%) (95 % CI)				IRS coverage (%) (95 % CI)				ITN or IRS (%) (95 % CI)			
Province	2006	2008	2010	2012	2006	2008	2010	2012	2006	2008	2010	2012
Central	48.4 (38.7–58.1)	51.0 (40.2–61.9)	74.0 (67.2–80.8)	55.7 (47.6–63.5)	12.0 (0.0–26.2)	19.5 (9.3–29.7)	13.0 (4.0–21.9)	25.7 (15.2–40.1)	55.5 (44.9–66.2)	57.2 (45.8–68.6)	77.7 (70.9–84.5)	65.2 (58.2–71.6)
Lusaka	26.1 (20.5–31.8)	55.4 (48.2–62.6)	49.9 (43.2–56.5)	55.4 (48.3–62.0)	12.2 (4.6–19.8)	30.7 (23.3–38.1)	34.8 (27.0–42.5)	12.3 (8.5–17.4)	34.3 (26.8–41.9)	66.6 (61.0–72.2)	65.2 (59.2–71.2)	60.0 (51.5–67.9)
Copperbelt	30.2 (24.4–35.9)	57.2 (51.0–63.5)	62.3 (56.3–68.3)	62.4 (53.4–70.6)	33.9 (19.6–48.2)	49.8 (40.2–59.4)	46.3 (39.3–53.4)	53.5 (46.4–60.4)	50.7 (38.2–63.2)	75.6 (70.4–80.7)	78.1 (73.3–83.0)	74.0 (66.0–80.7)
Eastern	33.5 (22.0–44.9)	74.8 (66.3–83.4)	76.1 (71.3–81.0)	87.6 (80.6–92.4)	1.8 (0.1–3.4)	0.6 (0.03–1.3)	14.0 (4.9–23.1)	33.7 (21.1–49.1)	34.2 (22.5–45.9)	74.8 (66.3–83.4)	77.8 (72.5–83.1)	90.4 (83.8–94.4)
Luapula	40.4 (28.7–52.0)	69.8 (62.7–76.9)	50.0 (43.2–56.5)	90.0 (85.6–93.1)	0.0 (0.0–1.1)	0.3 (0.0–0.7)	18.1 (4.9–31.3)	16.6 (8.4–30.2)	40.4 (28.7–52.0)	69.9 (62.9–77.0)	60.9 (51.8–70.0)	91.4 (87.7–94.0)
Northern	25.3 (16.6–33.9)	90.9 (85.2–96.7)	61.2 (52.5–69.9)	73.2 (62.6–81.7)	2.1 (0.0–5.7)	0.5 (0.0–1.3)	13.6 (3.1–24.0)	20.4 (9.9–37.4)	26.0 (17.1–34.9)	90.9 (85.2–96.7)	66.1 (56.2–76.1)	78.6 (71.1–84.5)
North-western	42.3 (31.9–52.8)	48.4 (25.6–71.2)	72.8 (60.8–84.8)	77.8 (68.6–84.9)	0.5 (0.0–1.4)	15.2 (0.0–33.7)	7.6 (0.0–17.6)	21.6 (7.9–46.9)	42.3 (31.9–52.8)	60.0 (35.1–85.0)	77.4 (70.1–84.7)	80.7 (73.9–86.1)
Southern	46.5 (32.6–60.5)	69.9 (60.8–79.0)	66.1 (55.4–76.7)	63.7 (57.5–69.5)	7.1 (0.0–19.6)	15.8 (2.8–28.9)	23.9 (9.7–38.2)	22.8 (12.6–37.7)	49.1 (33.1–65.1)	75.4 (66.9–83.9)	75.6 (64.5–86.6)	69.7 (61.7–76.7)
Western	67.0 (51.1–82.8)	33.8 (25.2–42.5)	74.7 (65.5–83.9)	51.9 (41.7–62.0)	1.3 (0.0–3.7)	0.0 (0.0–0.8)	28.6 (12.4–44.8)	12.4 (4.6–29.1)	67.0 (51.1–82.8)	33.8 (25.2–42.5)	79.3 (70.8–87.9)	56.0 (46.4–65.2)
National	37.8 (33.6–42.0)	61.0 (56.8–65.2)	64.3 (61.2 – 67.3)	68.1 (64.4–71.5)	9.6 (5.9–13.3)	16.0 (11.8–20.1)	24.0 (19.8–28.2)	25.9 (21.4–31.0)	43.3 (38.6–48.0)	67.3 (63.1–71.6)	72.9 (70.0–75.7)	73.9 (70.5–77.0)

Point estimates and confidence intervals account for survey design

The ITN-to-population surfaces produced as inputs for spatial prediction showed that this ratio increased overall across provinces from 2006 through 2010, but with substantial spatial heterogeneity, and increased dramatically in 2012 (Additional file 1: Figure S4). The number of ITNs per population increased overall between 2006 to 2008, but most notably in the north and east of the country. Between 2008 and 2010, the number of ITNs per population remained at similar levels in most of the west and south, but fell in the north, most notably in Luapula province. In 2012, this ITN-to-population ratio was highest in the northeast. IRS coverage was highly focal and higher near urban areas in 2006 and 2008, then increased nationally in 2010 and 2012 (Additional file 1: Figure S5).

While there were consistent declines in measured parasite prevalence across provinces from 2006 to 2008 during the peak transmission seasons in Zambia (national decline of child parasite prevalence from 22.4 % [95 % Confidence Interval (CI): 17.6–27.2 %] to 9.3 % [95 % CI: 7.0–11.6 %]), changes in prevalence between 2008 and 2010 at the province level were not uniform. Parasite prevalence increased significantly between 2008 and 2010 nationally (from 9.3 % to 15.9 %, *P* < 0.001), with quite large increases in Luapula (from 19.6 to 50.5 %, *P* < 0.001) and Eastern province (from 8.2 to 22.0 %, *P* < 0.001), and marginally in Northern (from 10.9 to 23.6 %, *P* = 0.052) (Table 2). Prevalence decreased slightly, but not significantly, in Northwestern (from 14.1 to 6.0 %, *P* = 0.150) and Southern Province (from 7.7 to 5.7 %). National prevalence in 2012 remained similar to 2010 (14.6 % [95 % CI: 11.9–17.7 %]); compared with 2010 there was a significant decrease in Luapula province (from 50.5 to 32.2 %, *P* < 0.05) and a non-significant decrease in Copperbelt province (from 12.1 to 4.6 %, *P* = 0.149), and slight increases in Northwestern (from 6.0 to 16.7 %, *P* = 0.063) and Western provinces (from 5.1 to 12.6 %, *P* = 0.098).

**Table 2:** Survey measured parasite prevalence in children 1–59 months. by year and province, Zambia 2006–2012

	Parasite prevalence (%) (95 % CI)			
Province	2006	2008	2010	2012
Central	31.0 (16.0–46.0)	5.6 (0.0–11.7)	9.2 (2.7–15.7)	8.6 (3.4–20.4)
Lusaka	0.0 (0.0–4.7)	1.2 (0.0–2.6)	0.0 (0.0–1.0)	0.0 (0.0–1.6)
Copperbelt	14.3 (5.2–23.4)	10.0 (4.9–15.0)	12.1 (6.0–18.2)	4.6 (1.3–15.4)
Eastern	23.8 (12.1–35.6)	8.2 (4.4–12.1)	22.0 (17.4–26.6)	20.4 (15.6–26.2)
Luapula	33.8 (23.0–44.5)	19.6 (12.5–26.7)	50.5 (41.2–59.8)	32.2 (22.1–44.4)
Northern	30.2 (18.9–41.6)	10.9 (6.0–15.7)	23.6 (9.5–37.7)	22.0 (15.7–30.0)
North-western	23.4^a^	14.1 (4.8–23.4)	6.0 (0.7–11.4)	16.7 (9.6–27.6)
Southern	15.5 (3.1–27.9)	7.7 (0.0–17.0)	5.7 (0.4–10.9)	8.5 (5.1–13.8)
Western	11.2 (1.6–20.9)	2.7 (0.0–5.9)	5.1 (1.2–8.9)	12.6 (6.0–24.7)
National	22.4 (17.6–27.2)	9.3 (7.0–11.6)	15.9 (12.2–19.6)	14.6 (11.9–17.7)

Point estimates and confidence intervals account for survey design

^a^data missing (prevalence estimated from rapid diagnostic test (RDT) field reports

In the full geostatistical model, children in the wealthiest households across the four surveys were less likely to have a parasite infection compared to the poorest (Adjusted odds ratio (AOR): 0.36, 95 % Bayesian Credible Interval (BCI): 0.24–0.52), as were individuals in areas of higher population density (> 1000/km-sq: AOR = 0.56, 95 % BCI: 0.35–0.89) and at higher altitudes (AOR = 0.19, 95 % BCI: 0.07–0.50) (Table 3), after controlling for climate. Individual household ownership of ≥ 1 ITN was associated with lower odds of infection (AOR = 0.74, 95 % BCI: 0.64–0.86). A higher SEA-level ITN density (ITNs per person) was not associated with malaria parasite infection in the full geostatistical model, but was inversely associated with infection in the prediction model (AOR = 0.42, 95 % BCI: 0.18–0.93). Higher SEA-level IRS coverage was significantly protective for child parasite infection (AOR = 0.30, 95 % BCI: 0.18–0.51). Higher rainfall preceding each survey was positively associated with odds of a parasite infection [standardized to 2 standard deviations (SDs)]: AOR = 2.04, 95 % BCI 1.38–3.00), as was a higher vegetation index (EVI) (standardized to 2 SDs: AOR = 1.98, 95 % BCI 1.48–2.65). Further details are presented in Additional file 1.

**Table 3:** Results of geostatistical models predicting malaria parasite prevalence in children 1–59 months, Zambia 2006–2012

Predictor		Full model		Prediction model	
		OR	95 % BCI	OR	95 % BCI
Age in years	< 1 (ref)				
	1–2	1.85	(1.48–2.32)		
	2–3	3.20	(2.57–3.97)		
	3–4	3.14	(2.53–3.90)		
	4–5	3.98	(3.19–4.95)		
Wealth	Poorest (ref)				
	Second	0.88	(0.74–1.04)		
	Third	0.76	(0.63–0.91)		
	Fourth	0.62	(0.50–0.77)		
	Richest	0.36	(0.24–0.52)		
Population size per km-sq	< 1000				
	At least 1000	0.56	(0.35–0.89)	0.45	(0.28–0.72)
HH ITN ownership	0				
	≥ 1	0.74	(0.64–0.86)		
Cluster ITN per person		0.62	(0.27–1.37)	0.42	(0.18–0.93)
Cluster IRS rate		0.30	(0.18–0.51)	0.25	(0.15–0.43)
Distance to nearest water (km)		0.98	(0.95–1.02)	0.98	(0.94–1.02)
Altitude (km)		0.19	(0.07–0.50)	0.17	(0.07–0.45)
Enhanced Vegetation Index	(2 SDs^a^)	1.98	(1.48–2.65)	2.09	(1.55–2.81)
Rainfall (mm)	(2 SDs)	2.04	(1.38–3.00)	2.03	(1.38–2.97)
Spatial variance		1.81	(1.35–2.39)	1.78	(1.33–2.35)

Odds ratio (OR) and 95 % Bayesian Credibility Interval (BCI) presented where relevant

^a^ *SD* standard deviation

Spatial prediction models were used to create continuous surfaces for predicted malaria parasite prevalence in children 1–59 months and ITN per person densities. SEA ITN density, SEA IRS coverage, population density, altitude, EVI, rainfall, and distance to nearest water body were used for producing the continuous parasite prevalence surface in 2006, 2008, 2010 and 2012 across Zambia. Out-of-sample validation of this spatial prediction model indicated decent model fit: the overall MPE was only -0.0125, indicating low bias, and the RMSE was 0.139, indicating decent accuracy.

**Fig. 3:**
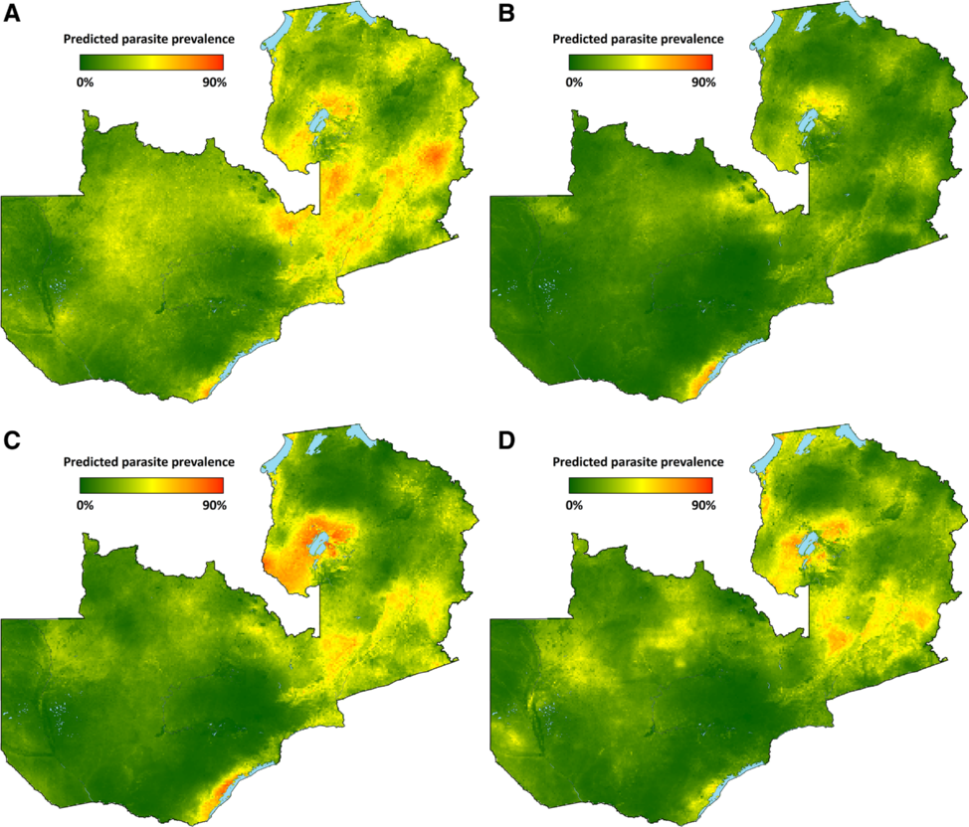
Predicted mean *Pf*PR_1–59_ surfaces for spatial prediction model in **a** 2006, **b** 2008, **c** 2010, and **d** 2012

**Fig. 4:**
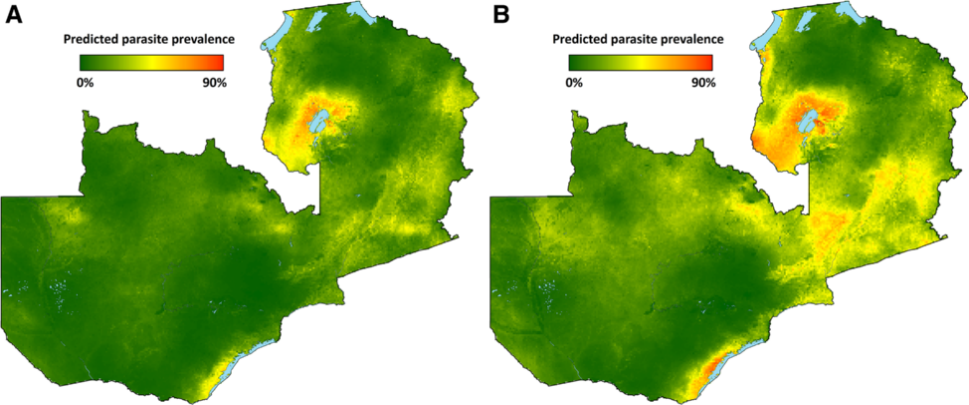
Predicted mean *Pf*PR_1–59_ surfaces for spatial prediction model under alternative prediction scenarios: **a** 2010 ITN/IRS, 2008 climate; **b** 2010 climate, 2008 ITN/IRS

Parasite prevalence risk surfaces show the highest risk in 2006 in Luapula, Northern, and Eastern provinces, and reduced risk across provinces in 2008 (Fig. 3). In 2010, resurgence of parasite prevalence was most notable in Luapula province and portions of Eastern province. From these predicted surfaces, population-adjusted parasite prevalence estimates were similar to the MIS estimates, with national and provincial level changes by survey year consistent with changes estimated by MIS tabulations (Table 4). Table 4 and Fig. 4 present alternative scenarios of climate and ITN coverage based upon altering prediction inputs.

**Table 4:** Modeled population-adjusted predicted prevalence (PA*Pf*PR_1–59_) by province and year, and predictions under constant climate or ITN scenarios, Zambia 2006–2012. Alternative predictions for 2010 were produced by including either the 2008 climate layers (for constant climate prediction) or the 2008 ITN and IRS layers (for constant vector control prediction)

	Annual predictions (%) (95 % BCI)				Alternative predictions for 2010 (%) (95 % BCI)	
Province	2006	2008	2010	2012	With constant climate	With constant vector control
Central	22.7 (5.0–52.4)	8.8 (1.3–27.1)	15.0 (3.2–36.4)	12.9 (2.7–33.2)	7.8 (1.3–23.4)	15.8 (3.4–37.6)
Lusaka	5.4 (0.7–16.5)	1.8 (0.2–5.8)	1.7 (0.2–5.4)	1.0 (0.1–3.7)	0.7 (0.1–2.7)	1.9 (0.2–5.7)
Copperbelt	17.3 (5.1–38.4)	14.8 (5.9–29.0)	13.2 (5.5–26.0)	6.0 (1.2–16.5)	7.1 (2.7–15.3)	12.8 (5.2–25.1)
Eastern	29.9 (8.6–60.0)	12.5 (3.2–30.7)	28.0 (10.1–54.1)	23.0 (7.7–47.4)	17.7 (5.4–39.3)	29.6 (10.9–56.1)
Luapula	31.6 (9.7–63.2)	19.9 (5.8–43.7)	35.1 (14.5–60.8)	32.8 (12.4–59.3)	27.5 (9.8–52.9)	35.4 (14.6–61.5)
Northern	26.8 (6.5–60.1)	14.4 (3.1–39.4)	17.3 (5.2–39.7)	19.7 (5.6–44.9)	12.3 (3.3–30.6)	17.0 (5.1–39.1)
North-western	22.3 (2.2–65.3)	12.7 (1.6–40.7)	16.0 (2.2–48.8)	17.6 (2.9–49.9)	9.6 (1.2–33.0)	17.1 (2.4–50.8)
Southern	11.4 (1.7–36.5)	7.1 (1.7–18.8)	9.6 (2.3–24.1)	7.7 (1.7–21.9)	6.1 (1.1–17.3)	9.5 (2.3–23.4)
Western	16.1 (2.2–47.3)	5.8 (0.6–22.1)	8.1 (1.0–27.9)	14.3 (2.3–42.1)	5.3 (0.6–19.8)	9.7 (1.3–31.9)
National	19.6 (4.6–46.4)	10.4 (2.6–26.8)	15.3 (4.9–33.7)	13.5 (3.7–31.7)	9.9 (2.7–24.2)	15.7 (5.0–34.3)

The model predicted an increase from 10.4 to 15.3 % (a 47 % relative increase) in national population-adjusted parasite prevalence (PA*Pf*PR_1–59_) between 2008 and 2010 across Zambia. Estimates of the expected prevalence if climate were constant between 2008 and 2010 indicated that the bulk of the change between 2008 and 2010 was due to climate factors. Based on spatial model predictions we estimated that a slight relative decrease (5 %) in parasite prevalence would have occurred between 2008 and 2010 if climate and environmental influences (rainfall and EVI) had remained at 2008 levels. Decreases in PA*Pf*PR_1–59_ between 2008 and 2010 were predicted for most provinces in this scenario, the exceptions being predicted increases, from 19.9 to 27.5 %, in Luapula, and from 12.5 to 17.7 % in Eastern province. Alternatively, holding ITN and IRS levels constant from 2008 to 2010 resulted in a predicted relative increase from 10.4 to 15.7 % (a 51 % relative increase) in parasite prevalence nationally based on climate alone, with the largest increases under this scenario in Luapula (from 19.9 to 35.4 %), Eastern (from 12.5 to 29.6 %) and Central (from 8.8 to 15.8 %) provinces.

## Discussion

We used a geostatistical model to assess the subnational associations between population-based measures of vector control coverage, inter-annual climate variability, and parasite prevalence in Zambia in 2006, 2008, 2010, and 2012 from the MIS in these years. In doing so, we were able to isolate the contributing effects of changes in ITN and IRS coverage and climate on changes in parasite prevalence at the subnational level in Zambia over this period, with a specific focus on changes that occurred between 2008 and 2010.

Rainfall, temperature suitability, and vegetation indices were all higher in 2006, 2010, and 2012, as compared with 2008. The measured malaria parasite prevalence across Zambia decreased from 22.4 % in 2006 to 9.3 % in 2008, then increased to 15.9 % in 2010 and 14.6 % in 2012. These changes in parasite prevalence were not uniform across the entire country and were mostly concentrated in specific provinces. Accounting for climate variations between 2006 and 2012 within a geostatistical framework, we show that the increase in parasite prevalence observed in several provinces between 2008 and 2010 corresponded both with decreases in household ITN coverage and with warmer and wetter conditions influencing the potential for underlying malaria transmission intensity, with a greater contribution due to climate variability. In geostatistical prediction models, we estimated that while the national increase in population-adjusted predicted parasite prevalence between 2008 and 2010 was from 10.4 to 15.3 % (a relative increase of 47 %), prevalence would have risen to 15.7 % (a relative increase of 51 %) if vector control was the same as in 2008. We estimate that while population-adjusted parasite prevalence increased relatively by almost 80 % from 2008 to 2010 in Luapula, prevalence would have increased by close to 40 % relatively due to lower ITN coverage alone. However, given the wide uncertainty in predicted prevalences, the credibility intervals for predictions overlapped, which limits our ability to make statistical conclusions about these results.

Although heterogeneity in ITN coverage over this period played a substantial role as ITN coverage fell in several of the provinces that experienced the greatest increase, these patterns do not completely explain the observed rebound. Higher community ITN density (ITNs per individual) and higher IRS household coverage were each significantly associated with reduced odds of parasite infection across all surveys, and coverage of each of these fell significantly in Northern and Luapula province between 2008 and 2010. Provinces where community vector control coverage in 2010 was at least as high in 2008 did not experience significant increases in infection prevalence, with the exception of Eastern province. In 2012, ITN coverage reached very high levels in Luapula and Eastern, but while prevalence dropped from 2010, it remained high. In Western province, ITN coverage fell in 2012, and this was associated with a more than doubling in parasite prevalence.

While IRS rates increased, the proportion of households with a newer ITN (< 2 years old) decreased dramatically in Northern, Luapula, and Eastern province between 2008 and 2010. This was likely the result of the staggered LLIN distribution campaigns that resulted in some provinces not receiving new nets until after 2010. This highlights the need for programs to work diligently at ensuring vector control coverage does not lapse in certain areas due to issues with net redistribution or spray efforts.

Climatic factors influencing malaria transmission, including rainfall, temperature suitability, and vegetation cover (EVI), were positively associated with malaria parasite prevalence in children across space and time. Overall, in Zambia, 2010 was wetter and hotter as compared to 2006 and 2008, with 2008 the driest and coolest of the time periods preceding each survey. This increase in rainfall and surface moisture in 2010 as compared to 2008 was most pronounced in areas experiencing the greatest increases in malaria parasite prevalence over this time period. Conditions in 2012 were similar to 2006, reinforcing that patterns in 2008 and 2010 were somewhat anomalous.

These changes in rainfall and temperature patterns may reflect regional effects of the *El Niño* Southern Oscillation (ENSO), as early 2010 was characterized by a moderately strong *El Niño* event, and early 2008 by a moderate *La Niña* event. However, the effects of the ENSO on the southern Africa region are varied, and likely dependent upon latitude [38]. Previous research found that *La Niña* years predict higher incidence in Botswana, which is similar ecologically to parts of southern Zambia [18]. Conversely, *El Niño* years have historically affected the northern part of the region [38]. This pattern may partially explain the rebound in the provinces in the northern part of country. Evidence of the influence of climate conditions favorable for malaria transmission in 2010 in southern Africa was not limited to Zambia. Similar trends were observed in subnational surveys in Malawi between 2005 and 2010 (PMI Malawi Impact Evaluation, personal communication). An increase in parasite prevalence in other countries in central Africa (e.g. Rwanda) may have also been influenced by a combination of intervention fall-back and climate factors. Trends in health facility data in Zambia and Malawi also coincide with a resurgence in malaria that began in 2009 and increased through 2010 [39].

Climate and ITN variables do not completely explain observed subnational changes in malaria parasite prevalence between 2008 and 2010. Immunological factors and other transmission dynamics between survey years may have influenced prevalence in ways not fully captured in geostatistical models. For example, older children in 2010 would have likely been less exposed to infectious mosquitoes in 2008 following the ITN distribution and a drier late-transmission season, and therefore possibly have weaker acquired immunity in 2010 than similarly aged children would have in 2008. They might therefore have been less able to clear infections or suppress parasite densities [40–42]. Other factors that must be considered in the resurgence in 2010 in some areas include ACT drug shortages and insecticide resistance. However, reported ACT levels were largely consistent between 2006 and 2010, and treatment seeking and treatment of fevers with an ACT has increased since 2006 [43]. Additionally, prevalence remained high in 2012, even as ACT coverage increased. As a result, these factors were not likely to have played a substantial role in increased prevalence over this period. Insecticide resistance may have contributed, but its role on a national scale is largely unknown, as studies have been very localized [10–12]. Mathematical modeling has furthermore suggested that the duration of the effects of LLIN provision may vary with transmission setting, such that the effective lifetime is shortened in higher transmission settings [44]. However, in our study ITNs at the community level and IRS at the household level were shown to be protective against malaria parasite infections across survey years, with no statistical evidence of ITN effectiveness decay as assessed by interaction terms between survey year and both household ITN ownership and community ITN density (non-significant interactions).

As with any cross-sectional study, this analysis was limited in its ability to adequately examine longitudinal trends. The utilization of repeated cross-sectional surveys allows only for examinations of contemporaneous association with limited causal inference [45]. Given that control with ITNs and IRS has been scaled-up nationwide, we lacked a true control group that would allow us to make stronger counterfactual arguments. We attempted to mitigate some of these limitations through the use of matching based upon residence status in non-spatial models and found associations similar to those in geostatistical models. Similarly, we approximated province level uncertainty in predictions by using the 2.5 and 97.5 % posterior quantiles at the pixel level. Given the known computational difficulties with producing aggregate-level uncertainty from geostatistical surfaces [46], we were not able in this study to incorporate the full pixel-level covariances. Additionally, further work is needed to parameterize potentially non-linear relationships between ITN coverage and parasite prevalence in a geostatistical framework.

The use of lagged climate variables to predict prevalence is confounded by the length of a malaria infection, which averages over 200 days without treatment [47]. However, as diagnostic and treatment capacity increased dramatically over this period, prevalence would have likely tracked incidence more closely as infections were cleared with more rapid effective treatment. Similarly, as transmission drops, as it did between 2006 and 2008, some children may lose acquired immunity, and therefore present with more symptomatic disease necessitating treatment. These effects would seem to bias toward shorter infections, which could potentially allow for more accurate estimation of climatic time lags with prevalence data. A strong relationship between climate and prevalence through time is supported by recent analyses of community-level surveillance data in Eastern province showing that the seasonality in prevalence closely tracks incidence [48].

## Conclusions

There is increasing effort to conduct repeated national surveys to generate data for malaria program evaluations. Without carefully considering and explicitly modeling confounding factors, especially climate, results of these surveys may lead to spurious conclusions of program effect or lack thereof, especially when assessing changes at the national level. In Zambia, it was surmised that ITNs began to fail after 2008 and contributed to increases in national infection prevalence by 2010. However, once one explicitly models changes in inter-annual climate variability at the subnational level, in conjunction with changes in ITN population coverage at the subnational level, we show that without the ITN coverage already in place in 2010, the malaria burden as measured by predicted parasite prevalence would have been worse in 2010 than actually observed. In doing so, this analysis represents a novel approach to applying geostatistical methods for malaria program evaluation. These results are important as malaria control programs attempt to interpret changes in prevalence and attribute them to program effort, or lack thereof, in the context of inter-annual climate variability. Subnational changes in malaria parasite prevalence are highly influenced by climate over short time scales and must be accounted for in assessing the effectiveness of malaria control programs.

## Additional file

Additional file 1: Supplementary information: Additional modeling results and maps of vector control coverage. (DOCX 1 mb)
