# Supplementary material for: The relative contribution of climate variability and vector control coverage to changes in malaria parasite prevalence in Zambia 2006–2012
Source: Parasit Vectors. 2016 Aug 5;9:431. doi: 10.1186/s13071-016-1693-0 (PMC4974721; doi:10.1186/s13071-016-1693-0)
Supplement: Additional file 1: — Supplementary information: Additional modeling results and maps of vector control coverage. (DOCX 1 mb) [file 13071_2016_1693_MOESM1_ESM.docx]

**Supplementary Information**

**Climate variable selection**

The best fitting lag for rainfall, measured as the linear relationship between 10-day rainfall and odds of parasitemia, was roughly seven weeks (Figure SI1), which is similar to previous research and a biologically plausible time lag between rainfall and onset of new infections [1]. This corresponded to a 20-day period of rainfall over late Feb and early March for the 2006 and 2008 survey, and a 20-day period of rainfall over mid to late February for the 2010 and 2012 surveys. The best lag for the temperature suitability index (TSI) was much closer to the survey date, in April for all survey years, and similarly for EVI, a 16-day period corresponding roughly to the start of each survey (Figures SI2 and SI3). Among all climatic covariates, the strongest bivariate relationship existed between EVI and odds of parasitemia.

**Figure S1.** Association between 10-day rainfall lags and odds of parasitemia. X-axis refers to monthly 10-day period.

**Figure S2.** Association between monthly temperature suitability index lags and odds of parasitemia.

**Figure S3.** Association between 16-day Enhanced Vegetation Index lags and log odds of parasitemia.

**Non-spatial multivariable models**

We first conducted multivariable random-effects logistic regression in a frequentist (non-Bayesian) setting to evaluate the association between vector control coverage, climate variability, and odds of malaria parasite infection. We examined the relationships between climate and vector covariates and odds of slide positivity in separate models for each year, and finally pooled all years. In the pooled model, we included survey year as a fixed effect to evaluate changes between surveys, while controlling for other factors in the model. We included survey SEA as a random effect. To assess the sensitivity of parameter estimates to various assumptions, we evaluated differences in parameter estimates between a population-average model using survey weights and one without. Additionally, we evaluated a model where we exactly matched [2] individuals by urban/rural status, transmission intensity (low: <10%, medium: 10-25%, high: >25%), and wealth (high/low), and included the matched strata as a random effect. We did not identify substantial differences in these sensitivity analyses; therefore we present only the results of random-effects models here.

After controlling for non-time-varying environmental factors and exact matching on residence, ownership of at least one ITN < 2 years old was associated with a 27% reduction in the odds of parasitemia [Adjusted odds ratio (AOR)=0.73, 95% CI 0.63-0.86] (Table SI1). At the cluster level, a one unit increase in the ITN to household member ratio was associated with a 67% reduction in odds of parasitemia (AOR=0.33, 95% CI 0.13-0.87), and a one unit increase in the SEA household IRS rate was associated with a 70% reduction in the odds of parasitemia (AOR=0.30, 95% CI 0.16-0.58) There was no significant interaction between ITN ownership or density and survey year or IRS and survey year.

Greater wealth status was associated with significant lower odds of parasitemia (AOR=0.37, 95% CI 0.26-0.58 for the wealthiest versus the poorest), as was living in a more populated area (>1000 persons/sq-km: AOR=0.39, 95% CI 0.23-0.67). Altitude (in kilometers) was associated with reduced odds of parasitemia (AOR=0.23, 95% CI 0.10-0.56). The indicator for survey year 2008 was significant (AOR=0.37, 95% CI 0.24-0.57).

Inclusion of time-varying environmental (climatic) factors improved model fit but did not greatly alter parameter estimates for household and community level vector control and other environmental factors. Higher lagged rainfall (mm) was significantly associated with increased odds of parasitemia (standardized to 2 Standard deviations: AOR=1.72, 95% CI 1.10-2.68), as was EVI (standardized to 2 Standard deviations: AOR=1.77, 95% CI 1.24-2.53). The temperature suitability index was not significant. After controlling for climate factors, none of the indicators for survey year were significantly associated with parasitemia.

**Table S1.** Non-spatial parasite prevalence model results.

| **Parameter** |  | **No climate variables** | | | **Climate variables included** | | |
| --- | --- | --- | --- | --- | --- | --- | --- |
|  |  | OR | 95%CI | p-value | OR | 95%CI | p-value |
| Age | <1 (ref) | 1 |  |  | 1 |  |  |
|  | 2  3  4  5 | 1.89  3.32  3.27  4.19 | (1.51-2.38)  (2.66-4.15)  (2.62-4.08)  (3.35-5.24) | <.001  <.001  <.001  <.001 | 1.89  3.31  3.26  4.17 | (1.50-2.37)  (2.65-4.13)  (2.61-4.06)  (3.33-5.22) | <.001  <.001  <.001  <.001 |
| Wealth | Poorest (ref) | 1 |  |  | 1 |  |  |
|  | Second | 0.87 | (0.73-1.03) | 0.104 | 0.87 | (0.73-1.03) | 0.116 |
|  | Third | 0.73 | (0.60-0.88) | 0.001 | 0.73 | (0.61-0.88) | <0.001 |
|  | Fourth | 0.61 | (0.49-0.76) | <.001 | 0.62 | (0.49-0.77) | <0.001 |
|  | Richest | 0.37 | (0.26-0.58) | <.001 | 0.39 | (0.27-0.60) | <0.001 |
| Population size per km-sq | <1000/km-sq (ref) | 1 |  |  | 1 |  |  |
|  | >1000/km-sq | 0.39 | (0.23-0.67) | 0.001 | 0.49 | (0.28-0.86) | 0.013 |
| Household ITN | None (ref) | 1 |  |  | 1 |  |  |
| >1 ITN |  | 0.73 | (0.63-0.86) | <.001 | 0.73 | (0.63-0.86) | <.001 |
| Cluster ITN per person |  | 0.33 | (0.13-0.87) | 0.025 | 0.38 | (0.15-0.99) | 0.047 |
| Cluster IRS rate |  | 0.30 | (0.16-0.58) | <.001 | 0.27 | (0.14-0.52) | <.001 |
| Distance to nearest water (km) |  | 1.00 | (0.96-1.04) | 0.918 | 1.00 | (0.96-1.04) | 0.911 |
| Altitude (km) |  | 0.23 | (0.10-0.56) | 0.001 | 0.35 | (0.13-0.93) | 0.035 |
| Temperature Suitability Index | (2 SDs) |  |  |  | 1.30 | (0.88-1.91) | (0.187) |
| Enhanced Vegetation Index | (2 SDs) |  |  |  | 1.77 | (1.24-2.53) | 0.002 |
| Rainfall (mm) | (2 SDs) |  |  |  | 1.72 | (1.10-2.68) | 0.017 |
| Province | Central | 1 |  |  | 1 |  |  |
|  | Copperbelt/NW | 2.75 | (1.56-4.82) | <.001 | 2.66 | (1.53-4.63) | 0.001 |
|  | Eastern | 2.24 | (1.27-3.93) | 0.005 | 1.82 | (1.04-3.21) | 0.037 |
|  | Luapula | 6.48 | (3.67-11.43) | <.001 | 5.04 | (2.84-8.92) | <.001 |
|  | Lusaka | 0.09 | (0.03-0.31) | <.001 | 0.14 | (0.04-0.46) | 0.001 |
|  | Northern | 2.82 | (1.62-4.91) | <.001 | 2.43 | (1.40-4.21) | 0.002 |
|  | Southern | 0.54 | (0.29-1.01) | 0.054 | 0.76 | (0.39-1.47) | 0.414 |
|  | Western | 0.44 | (0.22-0.88) | 0.019 | 0.61 | (0.31-1.29) | 0.209 |
| Year | 2006 | 1 |  |  | 1 |  |  |
|  | 2008 | 0.37 | (0.24-0.57) | <.001 | 0.67 | (0.37-1.19) | 0.168 |
|  | 2010  2012 | 0.74  1.34 | (0.48-1.13)  (0.75-2.41) | 0.163  0.320 | 0.72  1.49 | (0.46-1.13)  (0.83-2.66) | 0.152  0.178 |
| **AIC** |  | 7135 |  |  | 7126 |  |  |

**Bayesian geostatistical models**

Space-time Bayesian geostatistical models were parameterized as follows:

Y_ijk_~Ber(p_ijk_)

Logit(p_ijk_)= α + X_ijk_^T^ β + φ_jk_  + e_jk_

where Y_ijk_ is the binary outcome of parasitological status and p_ijk_ is the probability of parasitemia for child i at location j and time k when k=indicator for survey year (2006, 2008, 2010, 2012), α is the intercept, β represents the vector (β_1_,β_2_,…β_p_)^T^ of coefficients for each covariate represented in vector X_ijk_, φ_jk_ is a spatial random effect for each location, and e_jk_ is an exchangeable random effect for unobserved non-spatial variation.

In exploratory analysis, we constructed semi-variograms to evaluate spatial autocorrelation and estimate decay parameters. Spatial dependency was modeled as multivariate normal (φ_jk_~MN(0,Σ)), with a Matérn covariance function between locations s_i_ and s_i-1_ , where Σ (s_i_,s_i-1_)=σ^2^(κd(s_i_,s_i-1_)^v^Κ_v_(κd(s_i_,s_i-1_))/Γ(v)2^v-1^ where σ^2^ is the variance of the spatial process, d(s_i_,s_i-1_) is the Euclidean distance between locations s_i_ and s_i-1,_ κ is a scaling parameter, and Κ_v_ and Γ(v) are the modified Bessel function of second kind, order v, and Gamma function, respectively. The range of this spatial process (where spatial correlation becomes negligible) is defined as $\sqrt{8v}/\kappa$

For all geostatistical models, integrated nested Laplace approximation (INLA)[3] using the stochastic partial differential equation (SPDE)[4] approach was used for model fitting and prediction. All models were run using the INLA package in R [5]. Parasite infection status was modeled with a Bernoulli distribution. We modeled regression parameters with a normal prior with mean zero and inverse gamma variance; the prior on the variance was non-informative. Similarly the variance of the spatial random effect was given a vague log-Normal prior. The temporal process was defined by a first-order autoregressive function between survey years; the prior on the autoregressive parameter was set with a mean zero and a variance of 6. The covariates considered for inclusion in vector X_ijk_ included both environmental covariates (rainfall (lagged), temperature suitability index, enhanced vegetation index (EVI), altitude, distance to nearest water body, population density) and intervention coverage (HH ITN possession, community ITNs per person, distance from health facility, proportion of households with IRS per SEA). We examined separate models to assess the effect of vector control coverage and survey round in models with and without time-varying climatic variables. All models were assessed using the Deviance Information Criterion (DIC), where lower values indicate better model fit. We ultimately excluded temperature suitability index, distance from health facility, and province as these parameters were either collinear with other model parameters (altitude) or did not improve model fit. The final prediction space-time model had a DIC of 7067; a comparative non-spatial model with a temporal fixed effect had a DIC of 7231, indicating much better model fit provided by the space-time model.

The mean of the range parameter for spatial decay for the final prediction model was 1.27 (0.94-1.67) indicating a relatively high level of spatial autocorrelation, and the spatial variance was roughly four times the non-spatial variance.

**ITN and IRS coverage estimates**

To produce continuous ITN coverage estimates for each survey year the ratio of ITNs to persons in each SEA was modeled in a Bayesian geostatistical framework using a normal (Gaussian) distribution and with covariates urban/rural and distance to the district health office. As specified above, a Matérn covariance function was included to model spatial autocorrelation between SEAs and allow creation of a spatial prediction surface. Models were fit using INLA in R. For the final surfaces, predictions to each 5km x 5km grid cell covering Zambia (a total of 36,159 grid cells) were produced. IRS coverage was modeled in a similar fashion. Given the highly targeted nature of the IRS program, IRS survey data were less informative for predictions. To improve the predictions for IRS coverage, we included the number of IRS structures sprayed per district as a covariate in the prediction model. We converted the proportion of households sprayed with IRS per SEA using the logit transformation, and modeled the outcome of *logit* (IRS) for each survey year in a geostatistical model using INLA.

**Figure S4.** Predicted ITN to population density ratio for A) 2006, B) 2008, C) 2010, and D) 2012.


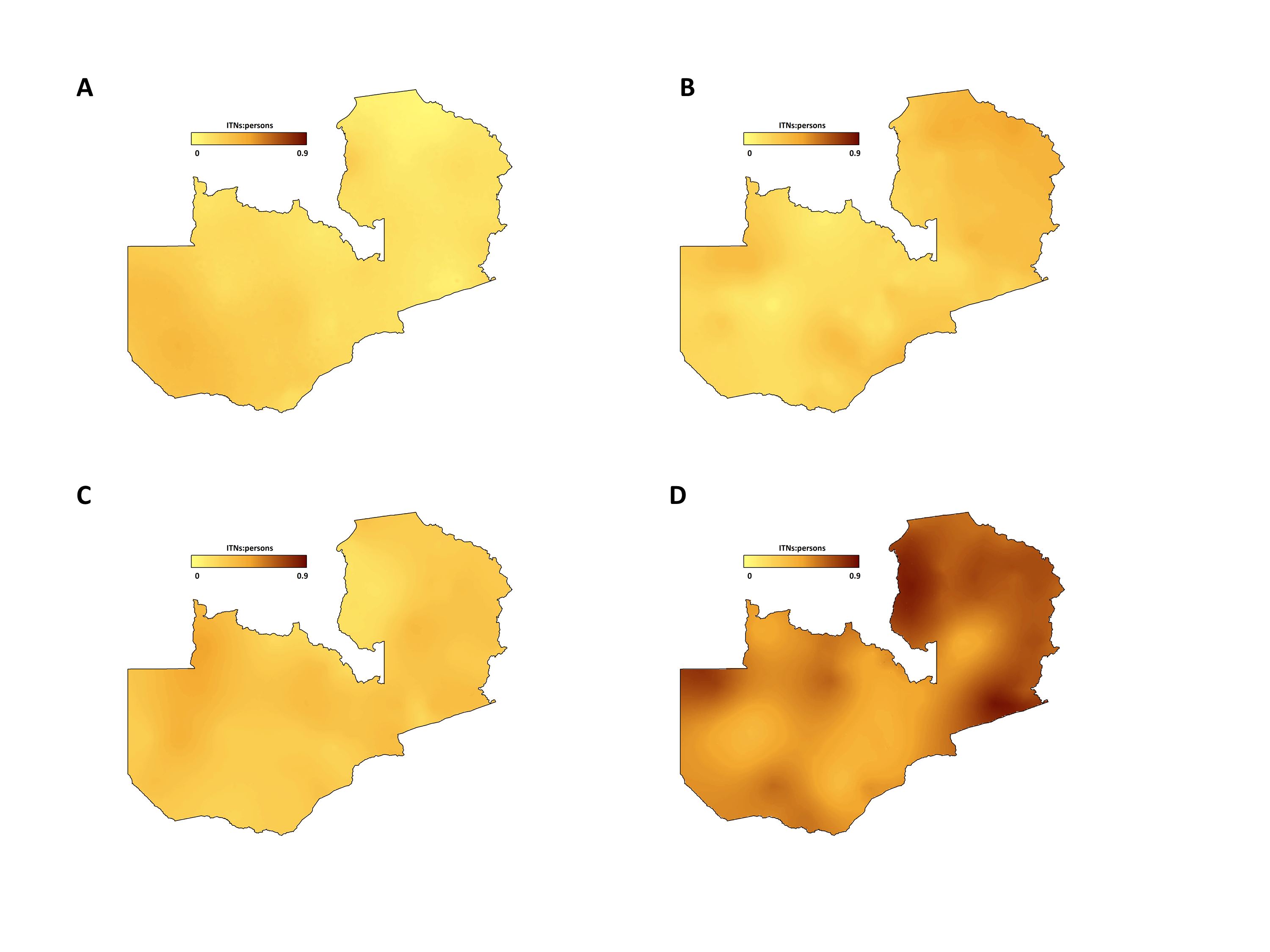


**Figure S5.** Predicted proportion of households with IRS for A) 2006, B) 2008, C) 2010, and D) 2012.


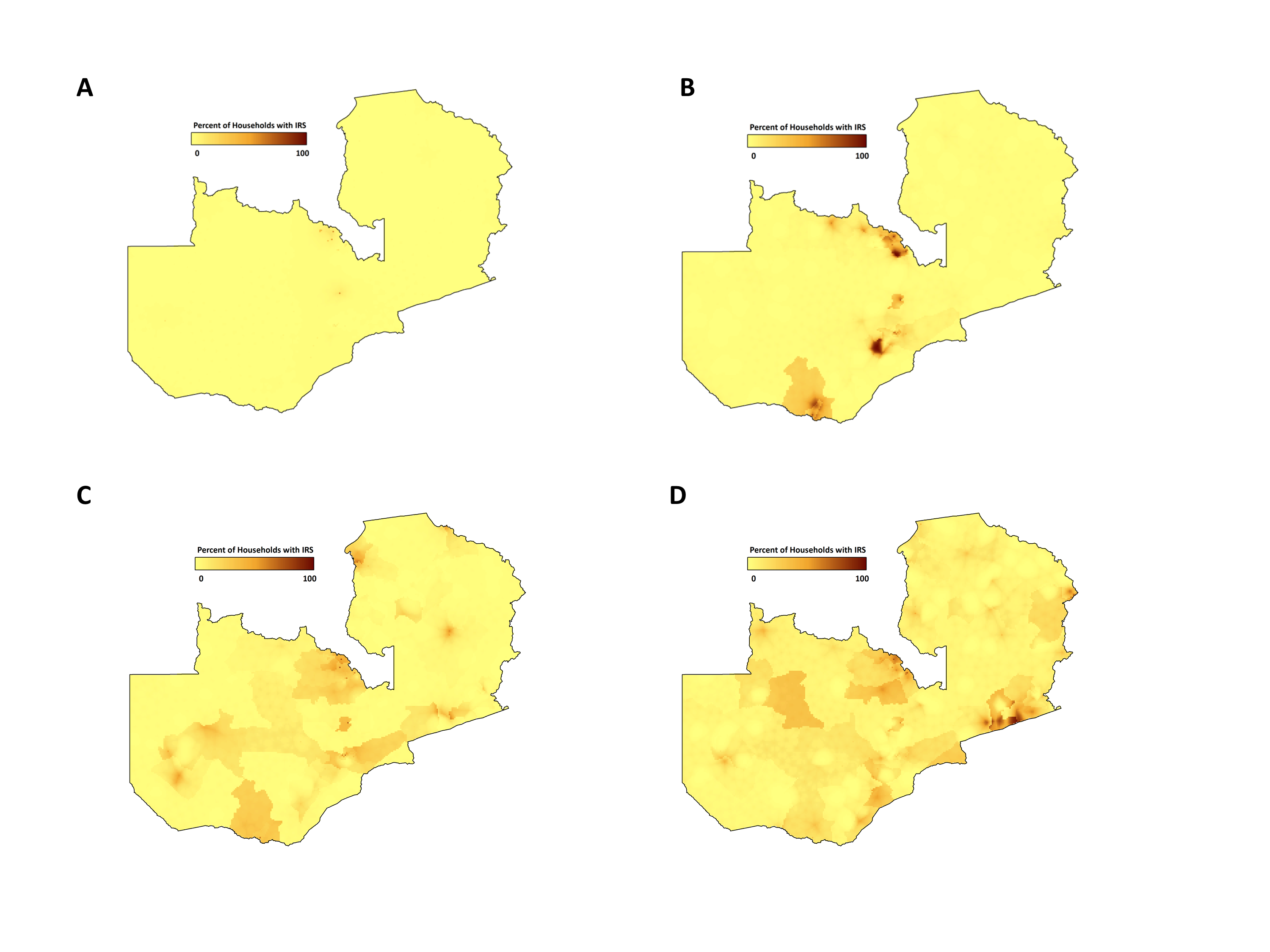


**References**

1. Teklehaimanot, H.D., et al., *Weather-based prediction of Plasmodium falciparum malaria in epidemic-prone regions of Ethiopia I. Patterns of lagged weather effects reflect biological mechanisms.* Malar J, 2004. **3**: p. 41.

2. Ho, D., et al., *Matching as Nonparametric Preprocessing for Reducing Model Dependence in Parametric Causal Inference.* Political Analysis, 2007(15): p. 199-236.

3. Rue, H., S. Martino, and N. Chopin, *Approximate Bayesian inference for latent Gaussian models using integrated nested Laplace approximations.* Journal of the Royal Statistical Society, Series B, 2009. **71**(2): p. 319-392.

4. Lindgren, F., H. Rue, and J. Lindstrom, *An explicit link between Gaussian fields and Gaussian Markov random fields: The SPDE approach (with discussion).* Journal of the Royal Statistical Society, Series B, 2011. **73**(4): p. 423-298.

5. *R-INLA*. 2016; Available from: [www.r-inla.org](http://www.r-inla.org).
